# Pancreatic Serous Cystadenomas Report of 8 Cases with a Mean Follow up of 7 Years

**DOI:** 10.1155/1996/92853

**Published:** 1996

**Authors:** Alejandro Fabiani, Gonzalo J. Delía, Roberto De Rosa, María T. Pombo, Oscar Molfino, Pedro Ferraina, Horace A. Laffaye

**Affiliations:** 1 Department of Surgery Centro de Educación Médica e Investigaciones clinicas (CEMIC) Buenos Aires Argentina; 2 Department of Pathology Centro de Educación Médica e Investigaciones clinicas (CEMIC) Buenos Aires Argentina; 3 Chairman Gastrointestinal Surgery University of Buenos Aires Hospital Buenos Aires Argentina; 4 Chairman Department of Surgery Norwalk Hospital Norwalk CT USA

## Abstract

Serous cystadenoma of the pancreas are rare tumors and have little or no malignant potential.

We report our experience in the management of eight casses of these tumors in the last 22 years. All the patients were women with a mean age of 59 years. All the cysts caused symptoms. Ultrasound and CTscan were useful in the diagnosis of the pancreatic cystic tumor out not in determining the nature of these lesions clear. FNA-biopsy was performed in 6 cases but in only one c se was the diagnosis confirmed. All tumors were resected. Four radical pancreatoduodenectomies, two distal pancreatectomies and two cystectomies were performed. Mean followup was 83.5 months. All patients are alive and with no signs of recurrence. Complications include an external pancreatic fistula, an acute cholangitis and a case of delayed gastric emptying. In all cases the histological diagnosis was serous cystadenoma of the pancreas. We conclude that resection of these tumors is mandatory although they are supposed to be benign, in order to avoid complications and because malignant transformations has been related to nonresective treatment.


*HPB Surgery*, 1996, Vol.9, pp.215–217
Reprints available directly from the publisher
Photocopying permitted by license only

© 1996 OPA (Overseas Publishers Association)
Amsterdam B.V. Published in The Netherlands
by Harwood Academic Publishers GmbH
Printed in Malaysia

### Pancreatic Serous Cystadenomas Report of 8 Cases with a Mean Follow up of 7 Years

ALEJANDRO FABIANI, * GONZALO J. DELÍA, * ROBERTO DE ROSA,*
MARÍA T. POMBO, ** OSCAR MOLFINO, * PEDRO FERRAINA, ***
and HORACE A. LAFFAYE ****

*Department of Surgery and ** Department of Pathology, Centro de Educación Médica e Investigaciones clínicas (CEMIC), Buenos Aires, Argentina
***Chairman Gastrointestinal Surgery, University of Buenos Aires Hospital, Buenos Aires, Argentina
**** Chairman Department of Surgery, Norwalk Hospital, Norwalk, CT, USA

*(Received 22 June 1994)*

Serous cystadenoma of the pancreas are rare tumors and have little or no malignant potential.

We report our experience in the management of eight casses of these tumors in the last 22 years. All the patients were women with a mean age of 59 years. All the cysts caused symptoms. Ultrasound and CT-scan were useful in the diagnosis of the pancreatic cystic tumor out not in determining the nature of these lesions clear. FNA-biopsy was performed in 6 cases but in only one c se was the diagnosis confirmed. All tumors were resected. Four radical pancreatoduodenectomies, two distal pancreatectomies and two cystectomies were performed. Mean followup was 83.5 months. All patients are alive and with no signs of recurrence. Complications include an external pancreatic fistula, an acute cholangitis and a case of delayed gastric emptying. In all cases the histological diagnosis was serous cystadenoma of the pancreas. We conclude that resection of these tumors is mandatory although they are supposed to be benign, in order to avoid complications and because malignant transformations has been related to nonresective treatment.

KEY WORDS: Pancreas  Cystic tumors  serous cystadenoma

Serous cystadenoma of the pancreas are rare tumors (9 to 13% of pancreatic cystic lesions) and have little or no malignant potential.[1,2,3].

With the improvement in imaging technology, several cystic lesions of the pancreas have been recognized (Table 1).[2] and the differential diagnosis is sometimes difficult.

We report our experience in the management of 8 patients with this kind of tumor in the last 22 years. Clinical presentation, diagnostic studies, surgical treatment and follow up is described with a brief review of the literature.

*Correspondence to*: Alejandro Fabiani, Departamento de cirugía, CEMIC, Las Heras 2900, Buenos Aires, Argentina.

**Table 1** Classification of Pancreatic Cystic Tumors (Warshaw *et al.*)[2].

| *Pancreatic Cystic Tumors* |
|---|
| –Serous cystadenoma (glycogen rich adenoma, microcystic adenoma) |
| –Mucinous cystadenoma (mucinous cystic neoplasm, macrocystic adenoma) |
| –Papillary cystic tumor (papillary and cystic neoplasm, solid and papillary neoplasm) |
| –Cystic islet cell tumor |
| –Pseudocyst |

### MATERIALS AND METHODS

The clinical records of eight patients with the diagnosis of serous cystadenoma of the pancreas surgically resected by our group in the last 22 years were reviewed. All pathology specimens were re-assesed and the diagnosis confirmed. The current situation of each patient was established by means of a full physical examination.

### RESULTS

All patients were women, with a mean age of 59 years (range 38–76 ys). All cysts caused symptoms, including epigastic pain, abdominal discomfort, nausea, weight loss and weakness (Table 2).

Ultrasound (US) was performed in all cases and CT-scan in the last six. In one case, before the advent of CT scanning, the tumor's origin was not clarified and in all the other patients these studies revealed a pancreatic cystic mass. In only one case serous cystadenoma was stated to be the most likely diagnosis and it was confirmed by fine needle aspiration (FNA) biopsy. FNA biopsy was performed under US-CT guidance in four cases and during the surgical procedure in two. No malignant cells were found, and in the case described above the diagnosis was made.

**Table 2** Clinical Manifestations of 8 patients with serous cystadenoma of the pancreas.

| *Clinical Manifestations* | |
|---|---|
| Epigastric pain or discomfort | 8 cases |
| Palpable abdominal mass | 4 cases |
| Nausea–Vomiting | 2 cases |
| Weight loss | 2 cases |
| Weakness | 1 case |

Four tumors were located in the head and the other four in the body of the pancreas. The mean size was 8.6 cm (range 3–20 cm) and there was no relationship between size and anatomical location.

All tumors were resected. Four radical pancreato-duodenectomies, two distal pancreatectomies and two cystectomies were performed.

Mean follow up was 83.5 months (range 12–264 months). All patients are alive and with no signs of recurence. One patient who underwent a radical pancreatoduodenectomy, developed an external pancreatic fistula, draining approximately 5 cc per 24 hours till it spontaneously closed 38 months after the resection. Another developed an episode of acute cholangitis secondary to a stone in the common bile duct and stenosis of the bilioenteric anastomosis 30 months after surgery. She was successfully treated endoscopically. finally, one patient developed delayed gastric emptying wich resolved spontaneously.

In all cases the histological findings were serous cystadenoma of the pancreas. Immunohistochemical findings are summarized in Table 3.

### DISCUSSION

Adloff in 1863, Fitz in 1900 and Malcolm in 1906 reported the earliest cases of pancreatic cystadenoma[4] but it was not until 1978 that Compagno and Ortel[1] fully described the differences between serous and mucinous cystic tumors of the pancreas.

Serous cystadenomas are considered to be benign as opposed to the mucinous cystadenomas which are malignant or premalignant tumors.[1] During the past few years multiple classifications of these tumors have appeared with great similarities in the nomenclature. (Table 1)[2]

Serous cystadenomas occur more frequently in females in the sixth and seventh decades, but ranging from 13 to 83 years of age.[1,2,5,6] They are usually quite large, averaging 10 cm in diameter[1,2]. Compagno and Ortel described them as made up of many tiny cysts lined by small cuboid cells containing glycogen but little or no mucin, forming a honeycomb pattern with no evidence of atypia.[2,7] Serous cystadenoma can occur in any segment of the pancreas and have also been described in ectopic pancreatic tissue.[6,8,9] In our series, the lesions developed in younger women and only two of them were larger than 10 cm.

The pathogenesis is unknown.[1,8,10] They are sometimes associated with extrapancreatic symptoms such as sterility, thymic dysfunciton, hypertension, diabetes mellitus, gallstones and Zollinger Ellison syndrome. No relationship has been established between the tumor and any of the above clinical conditions neither in our series nor in the literature.[8,11,12]

Pain is the most common symptom, being present in about 80% of cases, and is usually due to compression of contiguous structures. Other manifestations of the disease include weight loss, nausea and vomiting.

**Table 3** Immunohistochemical Findings in 8 cases of serous cystadenoma of the pancreas.

| *Immunohistochemical* | *Findings* |
|---|---|
| AE1–AE3 | ++++ Difuse |
| EMA | ++ Focal or Difuse |
| CEA | (-) |
| CHROMOGRANINE | (-) |

The rest of the patients are usually asymptomatic and in many cases the tumor is discovered during a routine clinical examination.[2,13] Occasionally they are found during work-up for other clinical problems.[14]

For a patient with a suspected cystadenoma of the pancreas, is it necessary to perform a surgical procedure for what is ultimately a benign condition? To us the answer is clear for although a spectrum of findings associated with serous cystadenoma have been described and Johnson et al. were able to correctly differentiate about 95% of serous cystadenoma from other cystic lesions of the pancreas[15] it is very difficult to differentiate a serous from a mucinous cystadenoma based only in the US-CT combination. Magnetic resonance imaging (MRI) is more expensive and has not demonstrated its superiority except for patients who cannot tolerate iodine-containing contrast material.[16]

FNA biopsy has been suggested as useful for confirming malignant tumors in unclear cases.[17] However it has several potential pitfalls, including sampling error and complications such as malignant cells spilling and seeding.[2,18] Analysis of cyst contents may be useful. Serous cystadenomas have no mucin[1] and positive immunostaining for the cytokeratins AE1 and AE3 or positive PAS reaction.[19]

In our experience, in only one case the US-CT findings suggested a serous cystadenoma and although we performed FNA biopsy in six cases (4 percutaneous with US-CT guidance and 2 intraoperatively) only in this one case was the diagnosis confirmed.

Considering our experience and the fact that 2 cases of serous cystadenocarcinoma have been reported recently[20,21] We conclude that a surgical procedure is necessary in order to confirm the diagnosis.

Another issue is which is the best surgical procedure for the resection of these tumors. The safety of the resection becomes an important consideration and is based primarily on the location of the tumor and the experience of the surgeon. Lesions in the body and tail of the pancreas are easily amenable to distal pancreatectomy while tumors of the head and of the uncinate process would require a Whipple procedure.[22] We followed this rule in six cases but enucleation was performed in two cases and no signs of relapse were found. We believe that resection, must be performed if possible, because complications such as hemorrhage, chronic pancreatitis, recurrent acute pancreatitis, obstructive symptoms and even malignant transformation have been related to nonresective treatment.[20,23]

### REFERENCES:

1. Compagno J, Ortel J. (1978) Microcystic adenomas of the pancreas (glycogen-rich cystadenomas) *American Journal of Clinical Pathology* **69**: 289–297.
2. Warshaw A, Compton C, Kent L.A, Cardenosa G, Mueller P. (1990) Cystic tumors of the pancreas. *Annals of Surgery* **212**: 432–445.
3. Bergmann L.S, Russell J.C, Gladstone A, Devers T. (1992) Cystadenoma of the pancreas. *American Surgeon* **58**: 65–71.
4. Strodel WE. (1981) Cystic neoplasms of the pancreas. In Pancreatic Diseases 1st edition edited by TL Dent, p 363–378, New York: *Grune & Stratton Inc.*
5. Corbally M.T, Mc Avena O, Urmacher C, Herman B, Shiu M. (1989) Pancreatic cystadenoma. *Archives of Surgery* **124**: 1271–1274.
6. Becker W, Welch R, Pratt H.P. (1965) Cystadenoma and cystadenocarcinoma of the pancreas. *Annals of Surgery* **161**: 475–478.
7. Kerlin D.L, Frey CF, Bodai B.I, Twoney P.L, Ruebner B. (1987) Cystic neoplasms of the pancreas. *Surgery, Gynecology & Obstetrics* **165**: 475–478.
8. Didolkar M.S, Malhotra Y, Holyoke E.D, Elias EG. (1975) Cystadenoma of the pancreas. *Surgery, Gynecology & Obstetrics* **140**: 925–928.
9. Urquijo S, Senosiain F.L, Ferrero A, Galvany A. (1983) Portal hypertension with cystadenoma of the pancreas. *Revista Española de Enfermedades Digestivas* **63**: 558–564
10. Elizondo E, Robles L, Arratibel A, Ruiz Diaz I. (1989) Cystadenoma of the pancreas: three new cases. *Revista Española de Enfermedades Digestivas* **75**: 593–596.
11. Taft D.A, Freeny P.C. (1981) Cystic Neoplasm of the Pancreas. *American Journal of Surgery* **142**: 30–33.
12. Hermoso S.C, Rosado R, Ramirez D, Ruiz J.J. (1989) Mucinous cyatadenoma of the pancreas. *Revista Española de Enfermedades Digestivas* **75**: 699–702.
13. Hodkinson D.J, Remine W.H, Welland LH. (1978) Pancreatic cystadenoma: a clinicopathological study of 45 cases. *Archives of Surgery* **113**: 443–446.
14. O'Delli M.L, Handler M.S, Wetzel L.H. (1991) Incidental detection of a microcystic adenoma of the pancreas. *Southern Medical Journal* **84**: 776–779.
15. Johnson C.D, Stephens D.H, Charboneau J.W. (1988) Cystic pancreatic tumors: CT and sonographic assessment. *American Journal of Radiology* **151**: 1133–38.
16. Minamin M, Itai Y, Ohtomo K *et al.* (1989) Cystic neoplasms of the pancreas: comparison of MR imaging with CT. *Radiology* **171**: 53–56.
17. Loser C, Folsch U. (1991) Value of imaging procedures in diagnosis of cystic pancreatic tumors. *Bildgebung* **58**: 177–81.
18. Jones E.C, Suen K.C, Grant D.R, Chan N.H. (1987) Fine-needle aspiration cytology of neoplastic cysts of the pancreas. *Diagnostic Cytopathology* **3**: 238–43.
19. Albores-Saavedra J, Angeles-Angeles A, Nadji M *et al.* (1987) Mucinous cystadenocarcinoma of the pancreas: morphologic and immunocytochemical observations. *American Journal of Surgical Pathology* **11**: 11–20.
20. George D.H, Murphy F, Michalski R, Ulmer B.G. (1989) Serous cystadenocarcinoma of the pancreas: a new entity? *American Journal of Surgical Pathology* **13**: 61–66.
21. Yoshimi N, Sugie S, Tanaka T, Aijin W, Bunai Y, Tatematsu A, Okada T, Mori H. (1992) A rare case of serous cystadenocarcinoma of the pancreas. *Cancer* **69**: 2449–53.
22. Pyke C.M, Van Heerden J.A, Colby T.V, Sarr MG, Weaver A.L. (1992) The spectrum of serous cystadenoma of the pancreas. Clinical, pathologic and surgical aspects. *Annals of Surgery* **215**: 132–139.
23. Sarles H, Cambon P, Choux R *et al.* (1988) Crhonic obstructive pancreatitis due to tiny (0.6 to 8 mm) benign tumors obstructing pancreatic ducts: report of three cases. *Pancreas* **3**: 232–237.

